# Functional variants of the 5-methyltetrahydrofolate-homocysteine methyltransferase gene significantly increase susceptibility to prostate cancer: Results from an ethnic Han Chinese population

**DOI:** 10.1038/srep36264

**Published:** 2016-11-03

**Authors:** Yuan-Yuan Qu, Shu-Xian Zhou, Xuan Zhang, Rui Zhao, Cheng-Yuan Gu, Kun Chang, Xiao-Qun Yang, Hua-Lei Gan, Bo Dai, Hai-Liang Zhang, Guo-Hai Shi, Yao Zhu, Ding-Wei Ye, Jian-Yuan Zhao

**Affiliations:** 1 Department of Urology, Fudan University Shanghai Cancer Center, Shanghai 200032, China; 2 Department of Oncology, Shanghai Medical College, Fudan University, Shanghai 200032, China; 3 The State Key Laboratory of Genetic Engineering and Collaborative Innovation Center of Genetics & Development, School of Life Sciences, Fudan University, Shanghai 200433, China; 4 Institutes of Biomedical Sciences, Fudan University, Shanghai 200032, China; 5 Department of Pathology, Fudan University Shanghai Cancer Center, Shanghai 200032, China

## Abstract

Aberrant DNA methylation has been implicated in prostate carcinogenesis. The one-carbon metabolism pathway and related metabolites determine cellular DNA methylation and thus is thought to play a pivotal role in PCa occurrence. This study aimed to investigate the contribution of genetic variants in one-carbon metabolism genes to prostate cancer (PCa) risk and the underlying biological mechanisms. In this hospital-based case-control study of 1817 PCa cases and 2026 cancer-free controls, we genotyped six polymorphisms in three one-carbon metabolism genes and assessed their association with the risk of PCa. We found two noncoding *MTR* variants, rs28372871 T > G and rs1131450 G > A, were independently associated with a significantly increased risk of PCa. The rs28372871 GG genotype (adjusted OR = 1.40, *P* = 0.004) and rs1131450 AA genotype (adjusted OR = 1.64, *P* = 0.007) exhibited 1.40-fold and 1.64-fold higher risk of PCa, respectively, compared with their respective homozygous wild-type genotypes. Further functional analyses revealed these two variants contribute to reducing *MTR* expression, elevating homocysteine and SAH levels, reducing methionine and SAM levels, increasing SAH/SAM ratio, and promoting the invasion of PCa cells *in vitro*. Collectively, our data suggest regulatory variants of the *MTR* gene significantly increase the PCa risk via decreasing methylation potential. These findings provide a novel molecular mechanism for the prostate carcinogenesis.

Prostate cancer (PCa) remains the most frequently occurring non-cutaneous solid malignancy in men from Western countries and represents the second leading cause of cancer-related death next to lung cancer^1^,^2^. The incidence of PCa in China, although lower than in developed countries, has increased remarkably partly due to the increasing life expectancy, dietary changes and Westernized lifestyle^3^. Recently, accumulated evidence suggests that genetic factors, such as genetic polymorphisms, may contribute to the etiology of PCa^4^,^5^,^6^.

Aberrant DNA methylation plays an essential role in prostatic tumorigenesis by stimulating proto-oncogenes and inactivating tumor suppressor genes^7^,^8^. Alteration of DNA methylation is often identified in PCa and is associated with PCa initiation by regulating gene expression and promoting chromosomal instability^9^,^10^,^11^. Several epigenetic mechanisms related to one-carbon metabolism involving DNA and histone methylation, DNA uracil misincorporation, and chromosomal rearrangements have been observed in PCa cells^12^,^13^. The one-carbon metabolism pathway is a complex network of interdependent reactions that facilitates the transfer of one-carbon units and ultimately provides various forms of precursors needed for DNA synthesis, repair and methylation. Numerous studies showed that one-carbon metabolism gene polymorphisms were associated with the balance of one-carbon metabolism and the genome-wide DNA methylation levels in breast cancer and colorectal cancer^14^,^15^,^16^,^17^. Moreover, our previous study identified several noncoding variants within one-carbon metabolism genes were able to impair the one-carbon metabolism balance and were associated with increased risk of birth defect^18^,^19^,^20^. However, whether those noncoding variants contributed to the occurrence of cancer remains unknown. Thus, investigating the roles of one-carbon metabolism gene variations in the cancer development is a topic of much current interest. Studies addressing the relationship between polymorphisms of one-carbon metabolism genes and the risk of various cancers, including colorectal cancer, breast cancer and malignant lymphoma, have yielded conflicting results^21^,^22^,^23^,^24^,^25^. Few studies have addressed the effects of one-carbon metabolism gene polymorphisms on the risk of PCa, and the results have been inconsistent^4^,^5^,^6^. Marchal and colleagues explored the association between polymorphisms of *MTHFR* (rs1801133, rs1801131), *MTR* (rs1805087) and *MTRR* (rs1801394) genes and risk of PCa in a Spain cohort and found *MTHFR* rs1801131 is clearly related to prostatic carcinogenesis^6^. Collin *et al.*^4^ investigated the effect of eight single nucleotide polymorphisms (SNPs), including *MTHFR* rs1801133, *MTHFR* rs1801131, *MTR* rs1805087, *MTRR* rs1801394, *MTHFD1* rs2236225, *SLC19A1/RFC1* rs1051266, *SHMT1* rs1979277 and *FOLH1* rs202676, on PCa risk in a meta-analysis and found no significant effects of any SNPs on susceptibility to PCa.

The accumulation of homocysteine causes elevated levels of its precursor S-adenosylhomocysteine (SAH), thus leading to aberrant DNA methyltransferase activity^26^,^27^,^28^,^29^,^30^. For this reason, the role of homocysteine removal gene polymorphisms in modulating the risk of PCa warrants further research. Furthermore, to the best of our knowledge, there are few studies of the possible correlation between genetic variations in the noncoding region of one-carbon metabolism genes and PCa risk have been published. The aim of the present study was to investigate the contribution of functional non-coding variants to the risk of PCa in a large-scale hospital-based case-control study, including *MTR* rs28372871, *MTR* rs1131450, *MTRR* rs326119 and *CBS* rs2850144^18^,^19^,^20^. Moreover, we assessed the effects of other extensively reported polymorphisms of genes in the one-carbon metabolism pathway, including *MTR* rs1805087 and *MTRR* rs1801394^4^,^6^,^31^, on the risk of PCa in our cohort.

## Results

### Characteristics of the study subjects

The demographic characteristics of the entire cohort are displayed in Table 1. The cases and controls were matched well by age, with a mean age of 66.7 and 66.9, respectively (*P* = 0.437). There were no significant differences in the distribution of age, BMI, hypertension, diabetes mellitus or cardiovascular disease between 1817 cases and 2026 controls. Among the case subjects, 605 (33.3%) patients were Gleason score ≥8, 586 (32.3%) patients had extracapsular extension, 352 (19.4%) patients had positive surgical margins and 154 (8.5%) patients had lymph node involvement.

### Association between one-carbon metabolism gene variants and PCa risk

The genotype frequencies and their associations with the risk of PCa are summarized in Table 2. The genotype frequencies among the controls were all in agreement with Hardy-Weinberg equilibrium (HWE) (all *P* > 0.05). The variants rs28372871, rs1131450 and rs1805087 within *MTR,* and rs326119 and rs1801394 within *MTRR*, were not in high linkage disequilibrium (Supplementary Tables 1 and 2). Significant differences in genotype distributions between cases and controls were observed for the non-coding *MTR* variants rs28372871 T > G (*P* = 3 × 10^−4^) and rs1131450 G > A (*P* = 2 × 10^−4^). The homozygous GG genotype of rs28372871 in the *MTR* gene promoter (crude OR = 1.44, 95% CI: 1.20–1.73, *P* = 3 × 10^−4^) and the homozygous AA genotype of rs1131450 in the 3′UTR of the *MTR* gene (crude OR = 1.77, 95% CI: 1.30–2.41, *P* = 2 × 10^−4^) were associated with a significantly increased risk of PCa compared with their respective homozygous wild-type genotypes. Furthermore, both the above-mentioned genotypes maintained their statistical significance in multivariate logistic regression analyses after adjusting for age, BMI, hypertension, diabetes mellitus and cardiovascular disease. Compared with the homozygous wild-types, there was a 1.40-fold increased risk of PCa associated with the *MTR* rs28372871 GG genotype (adjusted OR = 1.40, 95% CI: 1.15–1.71, *P* = 0.004) and a 1.64-fold increased risk associated with the *MTR* rs1131450 AA genotype (adjusted OR = 1.64, 95% CI: 1.17–2.31, *P* = 0.007). Nevertheless, we did not observe statistical evidence to support associations of the other SNPs with PCa risk (Table 2).

### Stratification analysis

Further stratified analyses were performed to investigate associations between the SNPs evaluated and PCa risk by recessive, dominant and log-additive genetic model, respectively (Table 3, Supplementary Tables 3 and 4). The multivariate logistic regression analyses revealed that the *MTR* rs28372871 GG genotype and rs1131450 AA genotype were associated with an increased risk of PCa, particularly in subgroups of Gleason score ≥8, positive extracapsular extension, positive seminal vesicle invasion and positive lymph node involvement in all three models, as supported by homogeneity tests (all *P* < 0.05). In addition, although increased risk was observed among subgroups of BMI ≥ 25 kg/m^2^ and lacking diabetes mellitus for patients carrying *MTR* rs28372871 GG genotype and among subgroups of age ≤68 years and without cardiovascular disease for those carrying *MTR* rs1131450 AA genotype in all three models, further homogeneity tests did not support any difference in the estimates of PCa risk between these strata.

### The promoter rs28372871 decreases *MTR* transcriptional activity in PCa cell lines and prostate tissues

**Figure 1:**
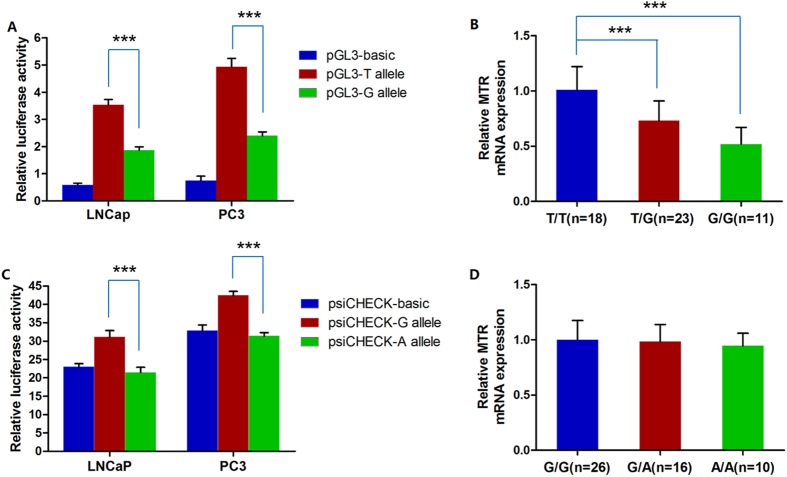
The *MTR* variants rs28372871 T > G and rs1131450 G > A down-regulate MTR expression at the levels of transcription and translation, respectively. (**A**) Luciferase expression is significantly decreased in the minor G allele construct compared with the major T construct in different cell types (47.2% reduction in LNCaP cells and 51.4% reduction in PC3 cells). (**B**) Quantitative real-time polymerase chain reaction (PCR) analysis of *MTR in vivo* mRNA expression in 52 human prostate tissue samples with different rs28372871 T > G genotypes. All values have been normalized to the level of *GAPDH*. (**C**) In luciferase assays, a plasmid construct with the minor A allele manifested luciferase activity significantly reduced by 31.1% compared with the major G allele in LNCaP cells. This value was 26.0% in PC3 cells. (**D**) Quantitative real-time polymerase chain reaction analysis of *MTR in vivo* mRNA expression in 52 human prostate tissue samples with different rs1131450 G > A genotypes. All values have been normalized to the level of *GAPDH*. Each value represents the mean ± SD of three independent experiments, and each experiment was performed in triplicate. ***indicates *P* < 0.001.

Because the rs28372871 T > G variant is located in the core promoter region of the *MTR* gene, we speculated that it might influence *MTR* transcription. Thus, we carried out *in vitro* luciferase reporter assays in the PCa cell lines LNCaP and PC3 and compared mRNA levels among different genotypes of the rs28372871 T > G variant in 52 human prostate tissue samples to assess the functional consequence of variant rs28372871 T > G on *MTR* transcriptional activity. The luciferase assay revealed that a plasmid containing the minor G allele provided significantly lower luciferase expression in comparison with the major A allele with a 47.2% reduction in LNCaP cells and a 51.4% reduction in PC3 cells (Fig. 1A). Data from human prostate tissue showed that samples with the rs28372871 TG and GG genotypes displayed 27.7% and 48.5% reductions in *MTR* gene expression, respectively, compared with the TT genotype (Fig. 1B). These consistent findings confirmed our hypothesis that the promoter rs28372871 T > G variant functionally reduces the transcription of *MTR*.

### The variant rs1131450 G > A reduces *MTR* expression

To investigate whether the rs1131450 G > A variant affects *MTR* expression, we performed *in vitro* luciferase reporter assays and assess the functional consequence of variant rs1131450 G > A on *MTR* transcriptional activity. The results of the transfection experiments showed that the plasmid construct carrying the mutant A allele manifested a 31.1% reduction in luciferase activity compared with that carrying the wild type G allele in LNCaP cells. There was a 26.0% reduction in PC3 cells (Fig. 1C). However, real-time PCR results showed that the variant rs1131450 was not related to *MTR* gene expression in human prostate tissue samples (Fig. 1D). Since the luciferase results indicated the protein level of target vector, these findings indicate that subjects carrying the *MTR* rs1131450 A allele might be at higher risk for PCa due to a reduction in MTR protein expression.

### *MTR* variants correlate with human plasma homocysteine concentration

**Figure 2:**
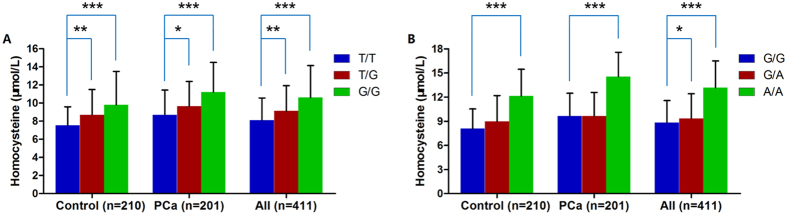
The functional *MTR* variants rs28372871 T > G and rs1131450 G > A correlate with human plasma homocysteine concentrations. (**A**) Homocysteine concentrations were significantly different between the groups with different rs28372871 T > G genotypes in the PCa cohort, the control cohort and the entire cohort. (**B**) Homocysteine concentrations were significantly different between the groups with different rs1131450 G > A genotypes in the PCa cohort, the control cohort and the entire cohort. The data shown are expressed as the mean ± SD. *indicates *P* < 0.05, **indicates *P* < 0.01, ***indicates *P* < 0.001.

MTR is responsible for the remethylation of homocysteine to methionine, which is essential for maintaining normal homocysteine concentrations. Because the rs28372871 and rs1131450 variants could reduce *MTR* expression and be correlated with plasma homocysteine concentration in adolescence, we assumed that those variants also determine homocysteine levels in the PCa susceptible population. To test this hypothesis, we explored the relationship between the two variants and plasma homocysteine levels in 201 PCa patients and 210 matched control subjects. Our results regarding the rs28372871 T > G variant showed that the TG and GG genotypes were significantly associated with elevated plasma homocysteine levels compared with the wild-type TT genotype in PCa and control subgroups as well as in combined analysis (all *P* < 0.05) (Fig. 2A). With regard to the rs1131450 G > A variant, carriers of the homozygous mutant AA genotype had the highest plasma homocysteine concentration, which was approximately 1.5-fold higher than those carrying the homozygous GG genotype in the PCa cohort, the control cohort and the entire cohort (Fig. 2B).

### Down-regulation of *MTR* elevates cellular homocysteine and SAH levels and reduces methionine and SAM levels in PCa cell lines

**Figure 3:**
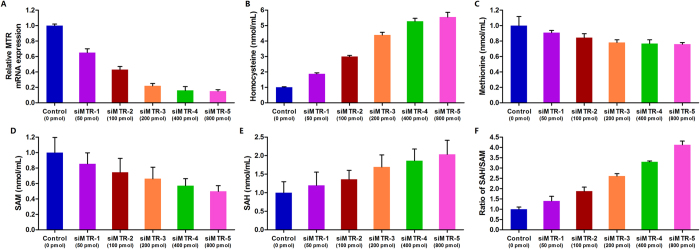
Down-regulation of *MTR* contributes to elevated cellular homocysteine and SAH levels, reduced methionine and SAM levels, and increased SAH/SAM ratio in PC3 cells. (**A**) Knockdown efficiency of gradient *MTR* siRNA was measured using quantitative real-time polymerase chain reaction. (**B**) Cellular homocysteine concentration after transfection with gradient *MTR* siRNA. (**C**) Cellular methionine level after transfected with gradient MTR siRNA. (**D**) Cellular SAM level after transfection with gradient *MTR* siRNA. (**E**) Cellular SAH level after transfection with gradient *MTR* siRNA. (**F**) Ratio of SAH/SAM after transfection with gradient *MTR* siRNA. Each value represents the mean ± SD of three independent experiments, and each experiment was performed in triplicate.

Furthermore, we detected cellular homocysteine, methionine, SAM and SAH concentrations in PC3 and LNCaP cells after gradient transfection with *MTR* siRNA. The *MTR* knockdown efficiency and metabolites quantification in PC3 cells were shown in Fig. 3. The results of quantitative real-time PCR, which was performed to determine the knockdown efficiency, showed that the expression of *MTR* was down-regulated in a dose-dependent manner (Fig. 3A). We observed elevated cellular homocysteine levels and reduced methionine levels with decreasing *MTR* expression (Fig. 3B,C). Moreover, decreased SAM concentration, increased SAH concentration, and an increased SAH/SAM ratio were observed when *MTR* expression decreased (Fig. 3D–F). Results obtained in LNCaP cells were consistent with those in PC3 cells (Supplementary Figure 2). These findings suggested that reduced *MTR* expression led to increased homocysteine levels and reduced cellular methylation potential.

### Down-regulation of *MTR* promotes cell invasion *in vitro*

**Figure 4:**
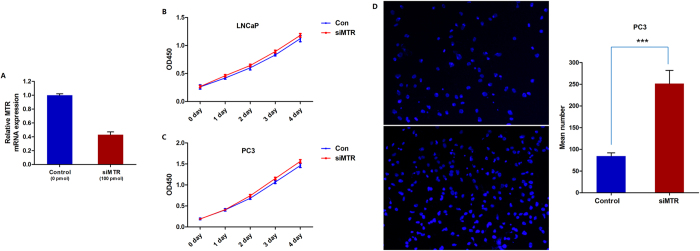
Down-regulation of *MTR* significantly increased PCa cell invasion but did not alter cell proliferation *in vitro*. (**A**) The knockdown efficiency of *MTR* siRNA was measured using quantitative real-time polymerase chain reaction. (**B**) Down-regulation of *MTR* did not alter LNCaP cell proliferation. (**C**) Down-regulation of *MTR* did not alter PC3 cell proliferation. (**D**) Down-regulation of *MTR* significantly increased invasion by PC3 cells. Each value represents the mean ± SD of three independent experiments, and each experiment was performed in triplicate.

Taking into consideration that the *MTR* genotype of risk led to nearly 50% reduction in *MTR* expression *in vivo* (Fig. 1B), LNCaP and PC3 cells with approximately 50% reduced *MTR* expression were constructed using siRNA (Fig. 4A). CCK-8 assays indicated that the approximately 50% reduction of *MTR* did not alter LNCaP or PC3 cell proliferation (Fig. 4B,C). However, in the PC3 cell invasion assay, we found that *MTR* knockdown significantly increased the number of invading cells following 24 hours of incubation compared with non-transfected cells (Fig. 4D and Supplementary Figure 1).

## Discussion

In the current large-scale hospital-based case-control study, we conducted a comprehensive analysis of a panel of genetic polymorphisms in 3 homocysteine removal genes involved in one-carbon metabolism and risk of PCa in 1817 cases and 2026 controls from an ethnic Han Chinese population. We found that two genetic variants in the regulatory regions of the *MTR* gene, rs28372871 T > G and rs1131450 G > A, were associated with a significantly increased risk of PCa. In previous study we proved that rs28372871 destroyed the transcription factor binding site in *MTR* promoter and rs1131450 enhanced the binding affinity of miRNA to the *MTR* 3′UTR^18^. In the current study, further functional analyses revealed that these two variants reduced the expression of the *MTR* gene *in vitro* and *in vivo*, elevated homocysteine and SAH levels, reduced methionine and SAM levels, increased the SAH/SAM ratio, and promoted the invasion of PCa cells *in vitro*. To the best of our knowledge, this is the first study to investigate the association between genetic variations in the noncoding region of one-carbon metabolism genes and risk of PCa.

MTR, one of the key enzymes in one-carbon metabolism, is responsible for the remethylation of homocysteine to methionine, thereby removing homocysteine. Consecutively, methionine is metabolized to yield SAM, which is the main methyl donor for methylation reactions. Studies addressing the relationship between *MTR* gene polymorphisms and risk of various cancers have yielded contradictory results. It was reported that *MTR* polymorphisms increase the risk of colorectal cancer^21^, breast cancer^22^, and malignant lymphoma^23^, but these findings have not been confirmed in other studies^24^,^25^. In a meta-analysis of one-carbon metabolism genes and risk of PCa, Collin *et al.* found that the *MTR* c.2756 A > G polymorphism was positively associated with PCa risk^4^. However, another study reported that there was no risk or statistically significant association between *MTR* polymorphism and PCa^5^. In the present study, we found that two noncoding *MTR* variants, rs28372871 T > G and rs1131450 905G > A, were independently associated with a significantly increased risk of PCa. The homozygous GG genotype of the variant rs28372871 in the promoter region (adjusted OR = 1.40, *P* = 0.004) and the homozygous AA genotype of the variant rs1131450 in the 3′UTR region (adjusted OR = 1.64, *P* = 0.007) exhibited a 1.40-fold and a 1.64-fold higher risk of PCa, respectively, compared with their respective homozygous wild-type genotypes in multivariate analyses. Furthermore, functional experiments revealed that both of the risk *MTR* allelic variants reduce *MTR* expression significantly. Decreased MTR expression results in elevated homocysteine levels with a simultaneous reduction of methionine production, consequently leading to an increased SAH/SAM ratio, which represents decreased methylation ability. Therefore, we inferred that the functional *MTR* variants rs28372871 and rs1131450 increase the risk of PCa via impairing methylation reactions considering that global DNA hypomethylation is a feature of prostatic tumorigenesis^32^,^33^,^34^.

MTRR catalyzes the regeneration of methylcobalamin, a cofactor of MTR, keeping MTR active. This is the first study to examine the association of the *MTRR* rs326119 polymorphism with the risk of PCa, and we report null results. Moreover, we did not observe an association between rs1801394, the most frequently studied *MTRR* polymorphism, and the risk of PCa. Previous studies that examined the *MTRR* rs1801394 polymorphism in relation to PCa risk generally showed null results, which is consistent with our results^4^,^6^.

CBS, as a rate-limiting enzyme, catalyzes the first irreversible step from homocysteine to cystathionine in the transsulfuration pathway. So far, few studies concerning the association between genetic polymorphisms in *CBS* and PCa risk have been published^5^. Kimura *et al.*^5^ investigated the association of *CBS* 844ins68 polymorphism and susceptibility to PCa and found no significant association. In this study, we found that the polymorphism rs2850144 C > G in the *CBS* gene promoter region was not associated with PCa risk. Recently, Zhang *et al.* observed an association between the *CBS* rs706209 polymorphism and clear cell renal cell carcinoma risk in the Chinese population^35^. Soon afterwards, Gallegos-Arreola and colleagues reported that the 844ins68 polymorphism in the *CBS* gene contributes significantly to breast cancer susceptibility in Mexican population^36^. Nevertheless, negative results have been observed with regard to different types of cancer^37^,^38^,^39^. Various factors, including differences in study design, study population and dietary assessment, could contribute to these inconsistent findings.

We acknowledge that there are certain limitations in the present study. Firstly, this hospital-based case-control study may have some selection and information biases, although these might be minimized by the age-matching between cases and controls as well as the adjustment for potential confounding factors in the statistical analyses. Secondly, although we investigated six SNPs in three key genes thought to be important in PCa risk in the one-carbon metabolism pathway, other potentially functional genetic variants may not have been included in the current study. Another potential limitation of this study is that the modest number of cases in the stratified analyses may limit the statistical power to examine the genetic variants with regard to the risk of PCa. Finally, the lack of data on folate intake and levels prohibits the evaluation of their effects on genetic variants in one-carbon metabolism genes and risk of PCa, thus limiting our conclusions. Notwithstanding these limitations, the results of the current study underscore that genetic variants in one-carbon metabolism genes may influence their function in one-carbon supply and subsequently result in elevated homocysteine levels and aberrant DNA methylation, thereby modifying PCa risk.

In summary, this large-scale hospital-based case-control study revealed that two functional variants in the regulatory regions of the *MTR* gene, rs28372871 T > G and rs1131450 G > A, were associated with a significantly increased risk of PCa by reducing *MTR* expression, elevating homocysteine and SAH levels, reducing methionine and SAM levels, increasing the SAH/SAM ratio, and promoting invasion by PCa cells. Our findings suggest that one-carbon metabolism plays a vital role in the etiology of PCa, and further investigation of gene-gene and gene-environment interactions in one-carbon metabolism is warranted.

## Materials and Methods

### Study population

This hospital-based case-control study recruited 1817 eligible patients with newly diagnosed PCa and 2026 matched cancer-free controls from genetically unrelated ethnic Han Chinese participants treated at Fudan University Shanghai Cancer Center from January 2008 to June 2015. All cases had histologically confirmed primary prostate adenocarcinoma assessed independently by two pathologists in routine diagnosis. All pathologic diagnoses were performed according to the WHO criteria for PCa. Cases who had malignancies other than primary PCa, had a family history of PCa and those who had radiotherapy or chemotherapy before enrollment were excluded. The tumor stage was determined and categorized according to the American Joint Committee on Cancer (AJCC) TNM classification system^40^. Histopathological grading was performed according to the Gleason score system. Patient clinicopathological characteristics including age, height, weight, serum PSA level at diagnosis, characteristics at surgery (tumor grade, tumor stage, surgical margin status and lymph node involvement) and comorbidities (hypertension, diabetes mellitus and cardiovascular disease) were extracted from the archival medical records.

The 2026 male cancer-free control subjects were frequency-matched to cases by age and geographic area and were recruited during the same period. Due to population-based PCa screening using PSA and digital rectal examination not being a routine practice in China, those who suffered from low urinary tract symptoms were advised to have serum PSA testing and a digital rectal examination, and subjects with serum PSA > 4 ng/mL with or without an abnormal digital rectal examination were excluded from the control group. The study was carried out in accordance with the ethical standards of the Helsinki Declaration II and approved by the Institution Review Board of Fudan University Shanghai Cancer Center. Written informed consent was obtained from each participant before any study-specific investigation was performed.

### SNP identification and genotyping

Genomic DNA was extracted from peripheral leukocytes by standard procedures using the Qiagen Blood DNA KIT (Qiagen Inc., Valencia, CA, USA). Polymorphisms in noncoding regions of the *MT*R (rs28372871, rs1131450), *MTRR* (rs326119) and *CBS* (rs2850144) genes were amplified by PCR. Selected SNPs, including polymorphisms in noncoding regions of the *MTR*, *MTRR* and *CBS* genes and the *MTR* rs1805087 and *MTRR* rs1801394 polymorphisms, were genotyped using SNaPshot analysis (ABI)^18^,^19^,^20^. Five percent of the genotyping results were validated using direct dye terminator sequencing of PCR products in accordance with the manufacturer’s protocol of the ABI Prism BigDye system (ABI, Foster City, CA, USA). The samples for sequencing and genotyping were run on an ABI 3730 automated sequencer and analyzed by SeqMan and Peakscan, respectively.

### Cell lines and cell culture

Human LNCaP and PC3 cell lines (The Cell Bank of Chinese Academy of Sciences, Shanghai) were cultured in RPMI 1640 media (Invitrogen, Carlsbad, CA) supplemented with 10% fetal bovine serum (Invitrogen). The cells were maintained in 5% CO_2_ at 37 °C and media was replaced every other day. Short tandem repeats genotyping and intermittent testing for androgen responsiveness (growth and androgen receptor activity) were performed to authenticate the cell lines. The genotypes of detected SNPs in these cell lines were detected and listed in Supplementary Table 5.

### Plasmid construction and luciferase reporter assay

The luciferase reporter plasmids were constructed as described before^18^. Briefly, 1304 bp *MTR* fragments from −1267 to +37 containing either T allele or G allele of rs28372871 were amplified by PCR from genomic DNA. After digestion with MluI and BglII, the PCR products were subcloned into the pGL3-Basic vector (Promega, Madison, WI, USA), in which the firefly luciferase gene was used as a reporter. To construct the *MTR* 3′UTR reporter plasmid, 878 bp fragments of the 3′UTR of the *MTR* gene containing either the G allele or A allele of rs1131450 were amplified from genomic DNA. The PCR products were subsequently digested using XhoI and BamHI and cloned into the 3′UTR of the *Renilla* luciferase gene of the psiCHECK-2 vector (Promega). The *Renilla* luciferase gene was used as a reporter, and its expression could be normalized to the firefly luciferase signal. The primers used are listed in Supplementary Table 6.

In luciferase reporter assay, 1 μg of each *MTR* reporter plasmid and 20 ng of the pRL-TK plasmid (Promega) as an internal control were transfected into LNCaP or PC3 cells. After 24 hours of transfection, cell lysates were collected and subjected to luciferase assay using Dual-Luciferase Reporter Assay System (Promega). Each luciferase assay was performed in triplicate, and three independent transfection experiments were carried out.

### Quantitative real-time PCR

We randomly collected 52 human prostate tissue samples from surgery of PCa patients and extracted total RNA using TransZol Up Plus Kit (Transgen Biotech, Beijing, China). After the total RNA was converted to cDNA using random hexamers, oligo primers and reverse transcriptase (Takara), the *MTR* mRNA were quantified by real-time PCR using the ABI Prism 7900 sequence detection system with *GAPDH* as an internal control. Each reaction was performed in triplicate. The primers utilized are listed in supplementary Table 6.

### siRNA transfection

*MTR* siRNA and mock siRNA negative control were chemically synthesized (Genepharma, Shanghai, China). For transfection, PC3 cells were seeded in six-well plates at 50–70% confluency. *MTR* siRNA was transfected with Lipofectamine 2000 (Invitrogen) according to the manufacturer’s instructions. At 48 hours after transfection, the cells were seeded for cell proliferation assays and invasion assays.

### Plasma and cellular one-carbon metabolite quantification

EDTA-plasma samples were randomly collected from 201 PCa patients and 210 cancer-free control participants, centrifuged immediately and frozen at −80 °C for quantifying the homocysteine levels. Plasma homocysteine levels were determined using the Axis® Homocysteine Enzyme Immunoassay (EIA) Kit (Axis-Shield, Norton, MA) according to the manufacturer’s instructions.

PC3 cells were transfected with 0 pmol, 50 pmol, 100 pmol, 200 pmol, 400 pmol or 800 pmol of *MTR* siRNA. After 48 hours of transfection, the cells were harvested for the detection of metabolite levels. Cellular homocysteine levels were determined using the Axis® Homocysteine Enzyme Immunoassay (EIA) Kit. The levels of S-adenosylmethionine (SAM) and SAH were detected with a SAM & SAH ELISA Combo Kit (Cell Biolabs, San Diego, CA). The concentration of methionine was quantified by high-performance liquid chromatography (HPLC) as previously reported^41^. Each test was repeated in triplicate, and the mean level was used for further analysis.

### Cell proliferation assay

Cell proliferation after transfection was measured using the Cell Counting Kit-8 (CCK-8) (Dojindo Laboratories, Kumamoto, Japan). Briefly, LNCaP and PC3 cells were seeded in 96-well plates. After incubation for 48 hours, CCK-8 solution (10 μl) was added to each well and incubated in a humidified CO_2_ incubator at 37 °C for 2 hours. Then, the absorbance of samples taken from each well was measured at 450 nm, on the basis of which the percentage of surviving cells in each treatment group was plotted relative to the untreated one.

### Cell invasion assay

Cellular invasion was measured using a Transwell chamber (Becton Dickinson, Franklin Lakes, NJ, USA) according to the manufacturer’s protocol. Briefly, after transfection, 1 × 10^5^ PC3 cells suspended in 100 μl F-12K medium without fetal bovine serum were seeded in the upper chamber of Transwell inserts with a pore size of 8 μm. A volume of 600 μl F-12K medium containing 10% fetal bovine serum as a chemoattractant was added to the lower chamber. Subsequent to a 24 hour incubation, the Transwell insert was washed with PBS, and cells on the upper surface of the insert were gently removed with a cotton swab. Invading cells (lower surface of the insert) were fixed with 4% paraformaldehyde (Sigma-Aldrich) for 15 min and were stained with DAPI. All assays were independently repeated in triplicate. Five random microscopic fields were evaluated for each insert.

### Statistical analysis

Body mass index (BMI, kg/m^2^) was calculated as weight in kilograms divided by height in meters squared and was categorized according to the WHO cut point for obese (BMI ≥ 25 kg/m^2^) in Asian populations^42^. Hardy-Weinberg equilibrium (HWE) for evaluation of genotype distribution in the control subjects was calculated using the goodness-of fit χ^2^ test, and *P* < 0.05 was considered deviated from the equilibrium. Univariate and multivariate unconditional logistic regression models were used to calculate crude and adjusted odds ratios (ORs) and 95% confidence intervals (CIs), respectively, to evaluate associations between the genotypes and PCa risk, with adjustments for age, BMI, hypertension, diabetes mellitus and cardiovascular disease in multivariate models. In addition, stratified analysis was performed to explore the association between the genotypes and risk of PCa among subgroups of age (≤68 vs. >68), BMI (<25 vs. ≥25), hypertension, diabetes mellitus, cardiovascular disease, Gleason score, extracapsular extension, seminal vesicle invasion, positive surgical margin and lymph node involvement. In the stratified analysis by age, patients were grouped using the median age (68 years old) as cutoff. The Chi-square-based Q test was performed to detect the homogeneity of associations between subgroups. For all statistical tests, a two-sided *P* < 0.05 was considered statistically significant. All statistical analyses were performed using SPSS (Statistical Package for the Social Sciences) software version 16.0 (SPSS Inc., Chicago, IL, USA).

## Additional Information

**How to cite this article**: Qu, Y.-Y. *et al.* Functional variants of the 5-methyltetrahydrofolate-homocysteine methyltransferase gene significantly increase susceptibility to prostate cancer: Results from an ethnic Han Chinese population. *Sci. Rep.* **6**, 36264; doi: 10.1038/srep36264 (2016).

**Publisher’s note:** Springer Nature remains neutral with regard to jurisdictional claims in published maps and institutional affiliations.

## Supplementary Material

Supplementary Information

## Figures and Tables

**Table 1:** Demographic and clinicopathological characteristics of 1817 PCa patients and 2026 controls included in the study.

Variables	Cases (n = 1817)	Controls (n = 2026)	*P*
Age (yr), mean ± SD	66.7 ± 7.2	66.9 ± 6.8	0.437
BMI (Kg/m2), n (%)			0.877
<25	1308 (72.0)	1463 (72.2)	
≥25	509 (28.0)	563 (27.8)	
Hypertension, n (%)			0.424
No	1054 (58.0)	1201 (59.3)	
Yes	763 (42.0)	825 (40.7)	
Diabetes mellitus, n (%)			0.926
No	1636 (90.0)	1826 (90.1)	
Yes	181 (10.0)	200 (9.9)	
Cardiovascular disease, n (%)			0.631
No	1660 (91.4)	1842 (90.9)	
Yes	157 (8.6)	184 (9.1)	
PSA (ng/mL), mean ± SD	28.5 ± 1.3	1.2 ± 0.3	< 0.001
Gleason score, n (%)			
≤6	289 (15.9)		
7	923 (50.8)		
≥8	605 (33.3)		
Pathological tumor stage, n (%)			
T2	1231 (67.7)		
T3a	160 (8.8)		
T3b	426 (23.4)		
Positive surgical margins, n (%)	352 (19.4)		
Lymph node involvement, n (%)	154 (8.5)		

**Table 2:** Associations between genetic polymorphisms in homocysteine removal genes and PCa risk in Han Chinese men.

Gene	SNP	Type	Genotype	Cases (n = 1817)	Controls (n = 2026)	*P*^HWE^	Crude OR (95% CI)	*P*	Adjusted OR (95% CI)^c^	*P*^a^
*MTR*	rs28372871	Promoter	TT	449 (24.7)	579 (28.6)	0.180	1.00	**3 × 10**^**-4**^	1.00	
			TG	910 (50.1)	1037 (51.2)		1.13 (0.97–1.32)		1.13 (0.96–1.33)	
			GG	458 (25.2)	410 (20.2)		**1.44 (1.20–1.73)**		**1.40 (1.15–1.71)**	**0.004**
			Dominant model				**1.22 (1.06–1.73)**	**0.007**	**1.20 (1.03–1.41)**	**0.020**
			Recessive model				**1.33 (1.14–1.55)**	**2 × 10**^**-4**^	**1.29 (1.10–1.53)**	**0.002**
			Log-additive model				**1.20 (1.09–1.31)**	**1 × 10**^**-4**^	**1.18 (1.07–1.30)**	**0.001**
	rs1131450	3'UTR	GG	1045 (57.5)	1267 (62.5)	0.120	1.00	**2 × 10**^**-4**^	1.00	
			GA	664 (36.5)	685 (33.8)		**1.18 (1.03–1.34)**		1.14 (0.98-1.32)	
			AA	108 (5.9)	74 (3.7)		**1.77 (1.30–2.41)**		**1.64 (1.17–2.31)**	**0.007**
			Dominant model				**1.23 (1.08–1.40)**	**0.001**	**1.19 (1.03–1.37)**	**0.017**
			Recessive model				**1.67 (1.23–2.26)**	**8 × 10**^**-4**^	**1.57 (1.12–2.20)**	**0.009**
			Log-additive model				**1.24 (1.11–1.38)**	**1 × 10**^**-4**^	**1.20 (1.06–1.35)**	**0.003**
	rs1805087	Nonsynonymous	AA	1481 (81.5)	1692 (83.5)	0.960	1.00	0.182	1.00	
		(Asp → Gly)	AG	316 (17.4)	319 (15.7)		1.13 (0.95–1.34)		1.11 (0.92–1.34)	
			GG	20 (1.1)	15 (0.7)		1.52 (0.78–2.99)		1.72 (0.83–3.53)	**0.190**
			Dominant model				1.15 (0.97–1.36)	0.102	1.14 (0.95–1.37)	**0.160**
			Recessive model				1.49 (0.76–2.92)	0.240	1.69 (0.82–3.47)	**0.150**
			Log-additive model				1.15 (0.99–1.34)	0.074	1.15 (0.97–1.36)	**0.100**
*MTRR*	rs326119	Intron-1	AA	896 (49.3)	944 (46.6)	0.880	1.00	0.220	1.00	
			AC	757 (41.7)	881 (43.5)		0.91 (0.79–1.03)		0.91 (0.79–1.05)	
			CC	164 (9.0)	201 (9.9)		0.86 (0.69–1.08)		0.86 (0.67–1.10)	0.310
			Dominant model				0.90 (0.79–1.02)	0.092	0.90 (0.78–1.04)	0.140
			Recessive model				0.90 (0.73–1.12)	0.345	0.90 (0.71–1.14)	0.390
			Log-additive model				0.92 (0.83–1.01)	0.087	0.92 (0.83–1.03)	0.130
	rs1801394	Nonsynonymous	AA	985 (54.2)	1111 (54.8)	0.420	1.00	0.831	1.00	0.89
		(Ile → Met)	AG	706 (38.9)	769 (38.0)		1.04 (0.91–1.18)		0.99 (0.85–1.14)	
			GG	126 (6.9)	146 (7.2)		0.97 (0.76–1.25)		0.93 (0.71–1.24)	
			Dominant model				1.03 (0.90–1.16)	0.697	0.98 (0.85–1.13)	0.770
			Recessive model				0.96 (0.75–1.23)	0.743	0.94 (0.71–1.23)	0.650
			Log-additive model				1.01 (0.91–1.12)	0.860	0.98 (0.87–1.09)	0.670
*CBS*	rs2850144	Promoter	CC	774 (42.6)	844 (41.7)	0.180	1.00	0.818	1.00	0.910
			CG	803 (44.2)	905 (44.7)		0.97 (0.84–1.11)		0.98 (0.84–1.14)	
			GG	240 (13.2)	277 (13.7)		0.94 (0.77–1.15)		0.96 (0.77–1.19)	
			Dominant model				0.96 (0.85–1.09)	0.556	0.97 (0.85–1.12)	0.710
			Recessive model				0.96 (0.80–1.16)	0.674	0.97 (0.79–1.18)	0.740
			Log-additive model				0.97 (0.89–1.06)	0.530	0.98 (0.88–1.08)	0.670

OR, odds ratio; 95% CI, 95% confidence interval.

^HWE^*P* value for the Hardy-Weinberg equilibrium test in control subjects.

^a^Adjusted for age, BMI, hypertension, diabetes mellitus and cardiovascular disease in multivariant logistic regression models.

**Table 3:** Stratified analysis for associations between genetic polymorphisms in homocysteine removal genes and PCa risk by recessive genetic model in Han Chinese men.

Variables	rs28372871 (cases/controls)		Adjusted OR^a^ (95% CI)	*P*	*P*^hom^	rs1131450 (cases/controls)		Adjusted OR^a^ (95% CI)		*P*	*P*^hom^	rs1805087 (cases/controls)		Adjusted OR^a^ (95% CI)	*P*	*P*^hom^
	TT/TG	GG				GG/GA	AA	AA/AG	GG							
Age (yr), median																
≤68	762/1031	253/274	1.21 (0.95–1.53)	0.120	0.294	959/1260	56/45	**1.77 (1.08–2.88)**		**0.022**	0.970	1006/1293	9/12	0.81 (0.29–2.23)	0.680	0.115
>68	597/585	205/136	**1.42 (1.04–1.96)**	**0.028**		750/692	52/29	1.53 (0.81–2.88)		0.180		791/718	11/3	3.14 (0.73–13.49)	0.110	
BMI (kg/m2)																
＜25	993/1166	315/297	1.22 (0.92–1.51)	0.074	0.193	1233/1410	75/53	1.49 (0.99–2.22)		0.053	0.768	1292/1452	16/11	1.79 (0.0.79–4.10)	0.160	0.631
≥25	366/450	143/113	**1.47 (1.07–2.01)**	**0.016**		476/542	33/21	1.83 (0.97–3.45)		0.061		505/559	4/4	1.30 (0.29–5.89)	0.730	
Hypertension																
No	801/954	253/247	1.14 (0.91–1.43)	0.250	0.199	994/1160	60/41	1.51 (0.95–2.42)		0.081	0.851	1045/1190	9/11	1.06 (0.39–2.87)	0.920	0.113
Yes	558/662	205/163	**1.45 (1.11–1.90)**	**0.006**		715/792	48/33	1.50 (0.88–2.57)		0.140		752/821	11/4	**4.17 (1.10–15.83)**	**0.026**	
Diabetes mellitus																
No	1224/1463	412/363	**1.35 (1.13–1.61)**	**9** **×** **10**^**−4**^	0.425	1539/1760	97/66	**1.55 (1.08–2.21)**		**0.017**	0.875	1620/1812	16/14	1.49 (0.69–3.23)	0.320	0.997
Yes	135/153	46/47	0.86 (0.48–1.54)	0.620		170/192	11/8	2.38 (0.82–6.87)		0.110		177/199	4/1	4.20 (0.27–64.20)	0.290	
Cardiovascular disease																
No	1254/1467	406/375	1.21 (0.92–1.46)	0.067	0.055	1558/1779	101/63	**1.80 (1.26–2.58)**		**0.001**	0.078	1646/1827	14/15	1.10 (0.50–2.44)	0.810	0.061
Yes	105/149	52/35	**2.32 (1.26–4.25)**	**0.006**		151/173	7/11	0.37 (0.10–1.36)		0.120		151/184	6/0	**NA (0.00-NA)**	**0.005**	
Gleason score																
≤7	932/1616	280/410	1.13 (0.93–1.38)	0.220	**0.017**	1153/1952	59/74	1.10 (0.73–1.66)		0.660	**0.037**	1204/2011	8/15	1.05 (0.41–2.71)	0.910	0.057
≥8	427/1616	178/410	**1.67 (1.31–2.14)**	**<0.0001**		556/1952	49/74	**2.83 (1.78–4.48)**		**<0.0001**		593/2011	12/15	**2.79 (1.13–6.87)**	**0.028**	
Extracapsular extension																
No	961/1616	270/410	1.03 (0.84–1.25)	0.780	**<0.0001**	1171/1952	60/74	1.20 (0.81–1.79)		0.370	**0.033**	1218/2011	13/15	1.42 (0.64–3.17)	0.390	0.835
Yes	398/1616	188/410	**2.08 (1.62–2.67)**	**<0.0001**		538/1952	48/74	**2.41 (1.51–3.87)**		**3** **×** **10**^**−4**^		579/2011	7/15	2.63 (0.86–8.05)	0.099	
Seminal vesicle invasion																
No	1074/1616	317/410	1.11 (0.92–1.34)	0.260	**<0.0001**	1321/1952	70/74	1.22 (0.83–1.79)		0.310	**0.022**	1375/2011	16/15	1.78 (0.83–3.82)	0.140	0.761
Yes	285/1616	141/410	**2.12 (1.60–2.82)**	**<0.001**		388/1952	38/74	**3.08 (1.83–5.18)**		**<0.0001**		422/2011	4/15	1.51 (0.39–5.89)	0.560	
Positive surgical margin																
No	1094/1616	371/410	**1.29 (1.08–1.54)**	**0.006**	0.837	1383/1952	82/74	**1.51 (1.05–2.16)**		**0.026**	0.300	1450/2011	15/15	1.43 (0.67–3.09)	0.360	0.601
Yes	265/1616	87/410	1.29 (0.93–1.81)	0.140		326/1952	26/74	1.79 (0.95–3.36)		0.078		347/2011	5/15	3.09 (0.81–11.71)	0.110	
Lymph node involvement																
No	1259/1616	404/410	**1.23 (1.04–1.47)**	**0.017**	**0.007**	1569/1952	94/74	**1.45 (1.02–2.05)**		**0.039**	0.133	1644/2011	19/15	1.75 (0.85–3.64)	0.130	0.198
Yes	100/1616	54/410	**1.96 (1.27–3.02)**	**0.003**		140/1952	14/74	**3.02 (1.39–6.57)**		**0.008**		153/2011	1/15	1.93 (0.18–21.10)	0.610	
**Variables**	**rs326119 (cases/controls)**		**Adjusted OR^a^ (95% CI)**	***P***	***Phom***	**rs1801394 (cases/controls)**		**Adjusted OR^a^ (95% CI)**		***P***	***Phom***	**rs2850144 (cases/controls)**		**Adjusted OR^a^ (95% CI)**	***P***	***Phom***
	**AA/AC**	**CC**				**AA/AG**	**GG**	**CC/CG**	**GG**							
Age (yr), median																
≤68	925/1181	90/124	0.93 (0.70–1.23)	0.600	0.701	938/1205	77/100	0.99 (0.73–1.35)		0.940	0.894	888/1140	127/165	0.99 (0.77–1.27)	0.920	0.593
>68	728/644	74/77	0.82 (0.53–1.26)	0.360		753/675	49/46	1.07 (0.63–1.80)		0.810		689/609	113/112	0.91 (0.64–1.30)	0.610	
BMI (kg/m2)																
＜25	1190/1319	118/144	0.89 (0.68–1.18)	0.430	0.905	1215/1365	93/98	1.07 (0.78–1.48)		0.660	0.197	1146/1275	162/188	0.92 (0.72–1.18)	0.530	0.979
≥25	463/506	46/57	0.91 (0.57–1.43)	0.670		476/515	33/48	0.70 (0.42–1.16)		0.160		431/474	78/89	1.07 (0.74–1.54)	0.710	
Hypertension																
No	959/1067	95/134	0.77 (0.56–1.05)	0.096	0.195	977/1122	77/79	1.17 (0.81–1.68)		0.400	0.153	907/1033	147/168	0.96 (0.73–1.26)	0.780	0.647
Yes	694/758	67/69	1.30 (0.88–1.94)	0.190		714/758	49/67	0.74 (0.47–1.18)		0.200		670/716	93/109	0.95 (0.67–1.35)	0.790	
Diabetes mellitus																
No	1481/1641	155/185	0.92 (0.72–1.18)	0.530	0.330	1519/1695	117/131	0.98 (0.74–1.31)		0.910	0.339	1421/1573	215/253	0.93 (0.75–1.16)	0.530	0.489
Yes	172/184	9/16	0.33 (0.10–1.15)	0.064		172/185	9/15	0.59 (0.20–1.74)		0.330		156/176	25/24	1.66 (0.81–3.38)	0.170	
Cardiovascular disease																
No	1512/1654	148/188	0.85 (0.66–1.09)	0.200	0.177	1543/1712	117/130	0.99 (0.74–1.32)		0.950	0.322	1441/1595	219/247	1.00 (0.80–1.24)	0.990	0.509
Yes	141/171	16/13	1.91 (0.73–4.99)	0.190		148/168	9/16	0.52 (0.17–1.54)		0.220		136/154	21/30	1.18 (0.55–2.50)	0.670	
Gleason score																
≤7	1104/1825	108/201	0.97 (0.74–1.29)	0.850	0.836	1126/1880	86/146	0.99 (0.72–1.36)		0.970	0.744	1058/1749	154/277	0.90 (0.71–1.14)	0.390	0.449
≥8	549/1825	56/201	0.80 (0.55–1.16)	0.230		565/1880	40/146	0.87 (0.57–1.34)		0.530		519/1749	86/277	1.09 (0.80–1.49)	0.570	
Extracapsular extension																
No	1129/1825	102/201	0.83 (0.63–1.10)	0.190	0.176	1146/1880	85/146	0.91 (0.67–1.25)		0.570	0.951	1069/1749	162/277	0.95 (0.75–1.21)	0.680	0.940
Yes	524/1825	62/201	1.11 (0.78–1.59)	0.560		545/1880	41/146	1.01 (0.66–1.55)		0.970		508/1749	78/277	0.96 (0.70–1.32)	0.810	
Seminal vesicle invasion																
No	1271/1825	120/201	0.89 (0.68–1.16)	0.370	0.351	1297/1880	94/146	0.92 (0.68–1.24)		0.590	0.642	1208/1749	183/277	0.95 (0.76–1.19)	0.650	0.917
Yes	382/1825	44/201	1.00 (0.66–1.52)	1.000		394/1880	32/146	1.05 (0.65–1.72)		0.830		369/1749	57/277	0.99 (0.69–1.42)	0.940	
Positive surgical margin																
No	1339/1825	126/201	0.84 (0.65–1.09)	0.200	0.257	1366/1880	99/146	0.89 (0.66–1.19)		0.410	0.594	1266/1749	199/277	0.98 (0.79–1.21)	0.830	0.389
Yes	314/1825	38/201	1.13 (0.71–1.79)	0.600		325/1880	27/146	1.28 (0.72–2.26)		0.410		311/1749	41/277	0.89 (0.58–1.36)	0.580	
Lymph node involvement																
No	1519/1825	144/201	0.86 (0.67–1.11)	0.240	0.100	1551/1880	112/146	0.91 (0.68–1.20)		0.500	0.310	1443/1749	220/277	0.98 (0.80–1.21)	0.870	0.936
Yes	134/1825	20/201	1.23 (0.66–2.26)	0.520		140/1880	14/146	1.51 (0.75–3.06)		0.260		134/1749	20/277	0.97 (0.54–1.74)	0.930	

OR, odds ratio; 95% CI, 95% confidence interval.

^hom^*P* value for homogeneity test using the χ^2^-based Q-test.

^a^Adjusted for age, BMI, hypertension, diabetes mellitus, and cardiovascular disease in multivariant logistic regression models.
