# Supplementary material for: Functional variants of the 5-methyltetrahydrofolate-homocysteine methyltransferase gene significantly increase susceptibility to prostate cancer: Results from an ethnic Han Chinese population
Source: Sci Rep. 2016 Nov 3;6:36264. doi: 10.1038/srep36264 (PMC5093691; doi:10.1038/srep36264)
Supplement: Supplementary Information [file srep36264-s1.pdf]

Supplementary file

Functional variants of the 5-methyltetrahydrofolate-homocysteine methyltransferase gene significantly increase susceptibility to prostate cancer: Results from an ethnic Han Chinese population

Running title: Functional variants of *MTR* increase susceptibility to PCa

Yuan-Yuan Qu, Shu-Xian Zhou, Xuan Zhang, Rui Zhao, Cheng-Yuan Gu, Kun Chang, Xiao-Qun Yang, Hua-Lei Gan, Bo Dai, Hai-Liang Zhang, Guo-Hai Shi, Yao Zhu*, Ding-Wei Ye*, Jian-Yuan Zhao*

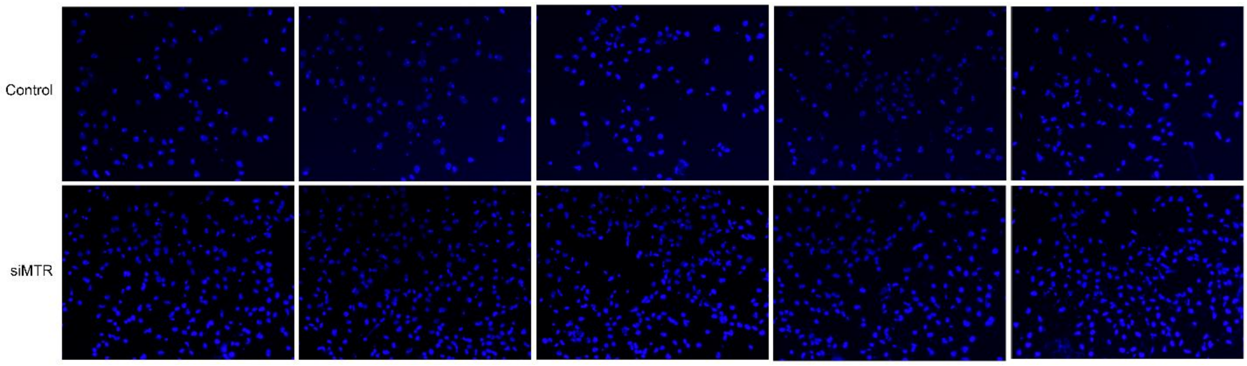

Supplementary Figure 1. Down-regulation of *MTR* significantly increased PCa cell invasion. Down-regulation of *MTR* significantly increased the number of invading PC3 cells. Each experiment was performed in triplicate and five random microscopic fields were evaluated for each insert.

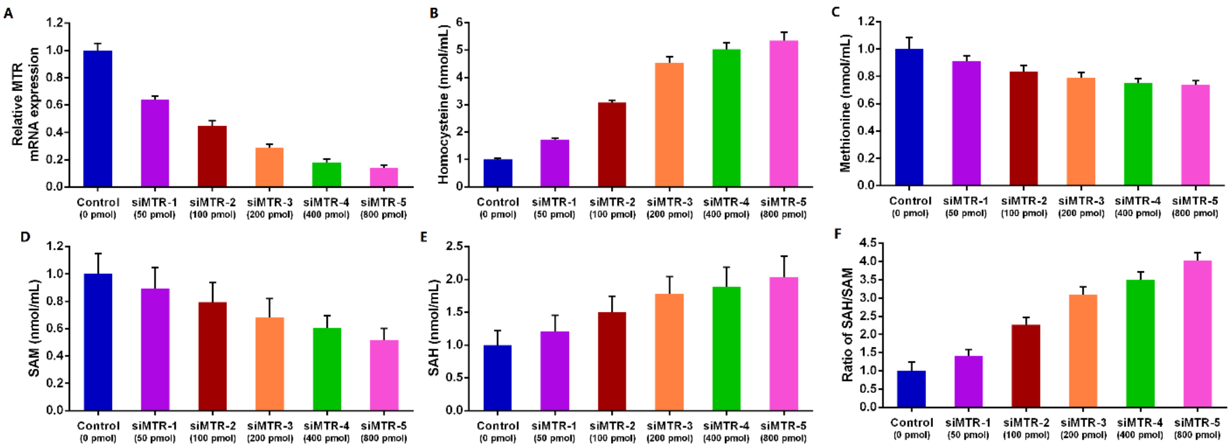

Supplementary Figure 2. Down-regulation of *MTR* contributes to elevated cellular homocysteine and SAH levels, reduced methionine and SAM levels, and increased SAH/SAM ratio in LNCaP cells. (A) Knockdown efficiency of gradient *MTR* siRNA was measured using quantitative real-time polymerase chain reaction. (B) Cellular homocysteine concentration after transfection with gradient *MTR* siRNA. (C) Cellular methionine level after transfected with gradient MTR siRNA. (D) Cellular SAM level after transfection with gradient *MTR* siRNA. (E) Cellular SAH level after transfection with gradient *MTR* siRNA. (F) Ratio of SAH/SAM after transfection with gradient *MTR* siRNA. Each value represents the mean ± SD of three independent experiments, and each experiment was performed in triplicate.

Supplementary Table 1. The linkage disequilibrium structure of *MTR* gene variants

	**D' statistic**				**R^2 statistic**		
Variants	rs28372871	rs1131450	rs1805087	Variants	rs28372871	rs1131450	rs1805087
rs28372871	.	0.265	0.646	rs28372871	.	0.022	0.046
rs1131450	.	.	0.235	rs1131450	.	.	0.002
rs1805087	.	.	.	rs1805087	.	.	.

Supplementary Table 2. The linkage disequilibrium structure of *MTRR* gene variants

	**D' statistic**			**R^2 statistic**	
Variants	rs326119	rs1801394	Variants	rs326119	rs1801394
rs326119	.	0.929	rs326119	.	0.137
rs1801394	.	.	rs1801394	.	.

Supplementary Table 3. Stratified analysis for associations between genetic polymorphisms in homocysteine removal genes and PCa risk by dominant genetic model in Han Chinese men

Variables	rs28372871 (cases/controls)		Adjusted OR[a] (95% CI)	*P*	P^{hom}	rs1131450 (cases/controls)		Adjusted OR[a] (95% CI)	*P*	P^{hom}	rs1805087 (cases/controls)		Adjusted OR[a] (95% CI)	*P*	P^{hom}
	TT	TG/GG				GG	GA/AA				AA	AG/GG			
Age (yr), median															
≤68	248/376	767/929	1.18 (0.95-1.48)	0.140	0.660	580/813	435/492	**1.27 (1.04-1.56)**	**0.018**	0.967	830/1085	185/220	1.08 (0.83-1.40)	0.580	0.507
>68	201/203	601/518	1.23 (0.92-1.65)	0.160		465/454	337/267	1.19 (0.92-1.56)	0.190		651/607	151/114	1.26 (0.90-1.78)	0.180	
BMI (kg/m2)															
<25	347/423	961/1040	1.12 (0.93-1.34)	0.230	0..069	779/916	529/547	1.10 (0.93-1.30)	0.250	0.052	1064/1223	244/240	1.16 (0.94-1.43)	0.180	0.753
≥25	102/156	407/407	**1.54 (1.12-2.12)**	**0.007**		266/351	243/212	**1.48 (1.13-1.94)**	**0.005**		417/469	92/94	1.08 (0.75-1.54)	0.680	
Hypertension															
No	274/344	780/857	1.11 (0.90-1.37)	0.320	0.291	599/748	455/453	1.17 (0.97-1.42)	0.100	0.768	859/1001	195/200	1.10 (0.86-1.41)	0.460	0.869
Yes	175/235	588/590	1.27 (0.98-1.64)	0.072		446/519	317/306	1.13 (0.89-1.43)	0.310		622/691	141/134	1.27 (0.94-1.71)	0.120	
Diabetes mellitus															
No	406/524	1230/1302	**1.21 (1.03-1.43)**	**0.024**	0.995	946/1140	960/686	1.15 (0.99-1.34)	0.064	0.474	1330/1523	306/303	1.15 (0.95-1.40)	0.160	0.217
Yes	43/55	138/145	1.29 (0.73-2.27)	0.380		99/127	82/73	1.47 (0.90-2.41)	0.130		151/169	30/31	1.34 (0.69-2.60)	0.390	
Cardiovascular disease															
No	415/525	1245/1317	1.18 (1.00-1.39)	0.053	0.387	953/1155	707/687	**1.19 (1.02-1.38)**	**0.026**	0.586	1353/1549	307/293	1.19 (0.98-1.44)	0.087	0.144
Yes	34/54	123/130	1.51 (0.81-2.82)	0.190		92/112	65/72	1.09 (0.63-1.87)	0.760		128/143	29/41	0.79 (0.41-1.56)	0.500	
Gleason score															
≤7	331/579	881/1447	1.05 (0.88-1.26)	0.570	**0.002**	736/1267	476/759	1.01 (0.86-1.20)	0.870	**0.001**	985/1692	227/334	1.17 (0.95-1.44)	0.150	0.758
≥8	118/579	487/1447	**1.64 (1.27-2.13)**	**0.0001**		309/1267	296/759	**1.64 (1.32-2.03)**	**<0.0001**		496/1692	109/334	1.17 (0.89-1.56)	0.270	
Extracapsular extension															
No	357/579	874/1447	0.94 (0.79-1.12)	0.500	**<0.0001**	773/1267	458/759	0.92 (0.78-1.08)	0.320	**<0.001**	1002/1692	229/334	1.16 (0.94-1.43)	0.170	0.883
Yes	92/579	494/1447	**2.27 (1.71-3.01)**	**<0.0001**		272/1267	314/759	**2.01 (1.62-2.51)**	**7×10^{-4}**		479/1692	107/334	1.26 (0.95-1.68)	0.120	
Seminal vesicle invasion															
No	383/579	1008/1447	1.02 (0.86-1.21)	0.790	**<0.0001**	844/1267	547/759	1.02 (0.88-1.20)	0.760	**<0.001**	1133/1692	258/334	1.18 (0.97-1.44)	0.100	0.924
Yes	66/579	360/1447	**2.41 (1.72-3.36)**	**<0.001**		201/1267	225/759	**1.99 (1.54-2.56)**	**<0.0001**		348/1692	78/334	1.24 (0.89-1.73)	0.200	

Supplementary Table 3 Continued. Stratified analysis for associations between genetic polymorphisms in homocysteine removal genes and PCa risk by dominant genetic model in Han Chinese men

Positive surgical margin															
No	358/579	1107/1447	**1.22 (1.03-1.45)**	**0.006**	0.623	847/1267	618/759	**1.16 (1.00-1.36)**	**0.005**	0.639	1197/1692	268/334	1.10 (0.90-1.33)	0.360	0.698
Yes	91/579	261/1447	1.11 (0.81-1.53)	0.510		198/1267	154/759	1.24 (0.93-1.66)	0.140		284/1692	68/334	**1.69 (1.17-2.45)**	**0.006**	
Lymph node involvement															
No	424/579	1239/1447	1.16 (0.98-1.36)	0.076	**0.016**	971/1267	692/759	1.13 (0.98-1.31)	0.100	**0.021**	1343/1692	320/334	**1.21 (1.00-1.46)**	**0.045**	**0.011**
Yes	25/579	129/1447	**1.99 (1.18-3.34)**	**0.006**		74/1267	80/759	**1.89 (1.26-2.84)**	**0.002**		138/1692	16/334	0.67 (0.36-1.24)	0.190	

Variables	rs326119 (cases/controls)		Adjusted OR[a] (95% CI)	P	P^{hom}	rs1801394 (cases/controls)		Adjusted OR[a] (95% CI)	P	P^{hom}	rs2850144 (cases/controls)		Adjusted OR[a] (95% CI)	P	P^{hom}
	AA	AC/CC				AA	AG/GG				CC	CG/GG			
Age (yr), median															
≤68	497/625	518/680	0.96 (0.81-1.13)	0.610	0.179	556/719	459/586	1.01 (0.86-1.19)	0.880	0.866	430/546	585/759	0.98 (0.83-1.16)	0.800	0.751
>68	399/319	403/402	0.78 (0.61-1.02)	0.067		429/392	373/329	0.97 (0.75-1.26)	0.840		344/298	458/423	0.92 (0.71-1.20)	0.540	
BMI (kg/m2)															
<25	646/670	662/793	0.86 (0.73-1.02)	0.078	0.382	710/809	598/654	1.01 (0.86-1.19)	0.910	0.696	555/614	753/849	0.99 (0.84-1.17)	0.920	0.630
≥25	250/274	259/289	1.00 (0.77-1.32)	0.980		275/302	234/261	0.92 (0.70-1.20)	0.540		219/230	290/333	0.94 (0.72-1.23)	0.660	
Hypertension															
No	527/542	527/659	**0.80 (0.67-0.97)**	**0.022**	0.110	554/672	500/529	1.14 (0.94-1.37)	0.180	0.041	436/514	618/687	1.12 (0.93-1.35)	0.240	0.077
Yes	369/402	394/423	1.02 (0.81-1.29)	0.840		431/439	332/386	0.80 (0.64-1.01)	0.060		338/330	425/495	0.82 (0.65-1.03)	0.094	
Diabetes mellitus															
No	803/852	833/974	0.91 (0.79-1.06)	0.230	0.584	893/1013	743/813	0.99 (0.85-1.14)	0.840	0.614	699/773	937/1053	1.02 (0.88-1.18)	0.810	0.290
Yes	93/92	88/108	0.72 (0.43-1.18)	0.190		92/98	89/102	0.89 (0.55-1.44)	0.630		75/71	106/129	0.71 (0.43-1.17)	0.180	
Cardiovascular disease															
No	819/843	841/999	0.87 (0.75-1.01)	0.070	0.098	900/1015	760/827	0.99 (0.86-1.15)	0.920	0.615	705/768	955/1074	0.99 (0.85-1.15)	0.880	0.740
Yes	77/101	80/83	1.72 (1.00-2.95)	0.050		85/96	72/88	0.77 (0.45-1.34)	0.360		69/76	88/108	1.06(0.62-1.81)	0.820	
Gleason score															
≤7	611/944	601/1082	0.84 (0.72-0.99)	0.039	0.262	645/1111	567/915	1.01 (0.86-1.19)	0.930	0.309	525/844	687/1182	0.96 (0.82-1.14)	0.660	0.458
≥8	285/944	320/1082	1.00 (0.81-1.24)	1.000		340/1111	265/915	0.92 (0.74-1.15)	0.470		249/844	356/1182	1.00 (0.80-1.24)	0.980	

Supplementary Table 3 Continued. Stratified analysis for associations between genetic polymorphisms in homocysteine removal genes and PCa risk by dominant genetic model in Han Chinese men

Extracapsular extension															
No	613/944	618/1082	0.87 (0.74-1.02)	0.089	0.612	654/1111	577/915	0.98 (0.84-1.16)	0.850	0.255	511/844	720/1182	1.04 (0.89-1.23)	0.600	0.251
Yes	283/944	303/1082	0.94 (0.76-1.17)	0.600		331/1111	255/915	0.93 (0.74-1.16)	0.500		263/844	323/1182	0.80 (0.65-1.00)	0.052	
Seminal vesicle invasion															
No	688/944	703/1082	0.89 (0.76-1.03)	0.120	0.842	738/1111	653/915	0.99 (0.85-1.16)	0.910	0.120	585/844	806/1182	1.00 (0.86-1.17)	0.990	0.464
Yes	208/944	218/1082	0.95 (0.74-1.22)	0.680		247/1111	179/915	0.86 (0.67-1.12)	0.260		189/844	237/1182	0.86 (0.67-1.11)	0.240	
Positive surgical margin															
No	722/944	743/1082	0.90 (0.78-1.05)	0.180	0.965	780/1111	685/915	1.01 (0.87-1.17)	0.940	0.135	622/844	843/1182	0.99 (0.85-1.15)	0.870	0.827
Yes	174/944	178/1082	0.82 (0.62-1.08)	0.160		205/1111	147/915	0.88 (0.66-1.17)	0.360		152/844	200/1182	0.93 (0.70-1.24)	0.630	
Lymph node involvement															
No	827/944	836/1082	0.88 (0.77-1.02)	0.094	0.274	898/1111	765/915	0.98 (0.85-1.13)	0.780	0.578	704/844	959/1182	0.99 (0.85-1.14)	0.840	0.483
Yes	69/944	85/1082	1.01 (0.68-1.50)	0.960		87/1111	67/915	0.96 (0.65-1.43)	0.860		70/844	84/1182	0.84 (0.56-1.26)	0.400	

OR, odds ratio; 95%CI, 95% confidence interval.

[a]Adjusted for age, BMI, hypertension, diabetes mellitus, and cardiovascular disease in multivariant logistic regression models.

^{hom}P value for homogeneity test using the χ^2-based Q-test.

Supplementary Table 4. Stratified analysis for associations between genetic polymorphisms in homocysteine removal genes and PCa risk by log-additive genetic model in Han Chinese men

	rs28372871		rs1131450		rs1805087	
Variables	Adjusted OR[a] (95% CI)	*P*	Adjusted OR[a] (95% CI)	*P*	Adjusted OR[a] (95% CI)	*P*
Age (yr), median						
≤68	1.14 (0.99-1.32)	0.061	**1.28 (1.08-1.52)**	**0.005**	1.05 (0.83-1.34)	0.680
>68	**1.23 (1.02-1.49)**	**0.027**	1.20 (0.96-1.50)	0.110	1.30 (0.95-1.78)	0.100
BMI (kg/m2)						
<25	1.13 (1.00-1.27)	0.045	1.13 (0.98-1.30)	0.093	1.17 (0.96-1.43)	0.120
≥25	**1.37 (1.12-1.66)**	**0.002**	**1.43 (1.13-1.79)**	**0.002**	1.08 (0.77-1.51)	0.650
Hypertension						
No	1.09 (0.96-1.25)	0.190	1.18 (1.00-1.39)	0.044	1.09 (0.86-1.37)	0.480
Yes	**1.26 (1.07-1.48)**	**0.006**	1.15 (0.94-1.39)	0.170	1.32 (1.00-1.74)	0.050
Diabetes mellitus						
No	**1.20 (1.08-1.33)**	**7×10^{-4}**	**1.17 (1.03-1.33)**	**0.016**	1.15 (0.96-1.38)	0.120
Yes	1.05 (0.74-1.49)	0.800	1.46 (0.98-2.19)	0.063	1.38 (0.75-2.55)	0.300
Cardiovascular disease						
No	1.15 (1.03-1.27)	0.011	**1.22 (1.07-1.38)**	**0.002**	1.16 (0.97-1.39)	0.100
Yes	1.62 (1.11-2.38)	0.012	0.93 (0.60-1.45)	0.750	1.04 (0.58-1.87)	0.890
Gleason score						
≤7	1.07 (0.95-1.20)	0.280	1.02 (0.89-1.18)	0.770	1.15 (0.94-1.40)	0.170
≥8	**1.47 (1.26-1.71)**	**<0.0001**	**1.63 (1.37-1.95)**	**<0.0001**	1.23 (0.96-1.58)	0.110
Extracapsular extension						
No	0.98 (0.88-1.10)	0.780	0.96 (0.84-1.11)	0.590	1.16 (0.95-1.40)	0.140
Yes	**1.81 (1.54-2.14)**	**<0.0001**	**1.84 (1.53-2.21)**	**<0.0001**	1.29 (0.99-1.68)	0.066
Seminal vesicle invasion						
No	1.05 (0.94-1.17)	0.410	1.04 (0.91-1.19)	0.550	1.19 (0.99-1.43)	0.064
Yes	**1.86 (1.54-2.25)**	**<0.001**	**1.89 (1.53-2.32)**	**<0.0001**	1.23 (0.90-1.68)	0.190
Positive surgical margin						
No	**1.19 (1.07-1.32)**	**0.002**	**1.17 (1.03-1.33)**	**0.014**	1.10 (0.92-1.32)	0.290
Yes	1.15 (0.93-1.40)	0.190	1.26 (0.99-1.61)	0.060	1.67 (1.19-2.35)	0.004

Supplementary Table 4 Continued. Stratified analysis for associations between genetic polymorphisms in homocysteine removal genes and PCa risk by log-additive genetic model in Han Chinese men

Lymph node involvement						
No	1.14 (1.03-1.27)	0.011	**1.14 (1.01-1.29)**	**0.035**	**1.21 (1.02-1.44)**	**0.027**
Yes	**1.67 (1.25-2.23)**	**4×10^{-4}**	**1.83 (1.32-2.54)**	**3×10^{-4}**	0.72 (0.40-1.28)	0.250

	rs326119		rs1801394		rs2850144	
Variables	Adjusted OR[a] (95% CI)	*P*	Adjusted OR[a] (95% CI)	*P*	Adjusted OR[a] (95% CI)	*P*
Age (yr), median						
≤68	0.96 (0.85-1.09)	0.530	1.01 (0.88-1.14)	0.930	0.99 (0.87-1.11)	0.820
>68	0.83 (0.68-1.02)	0.070	0.99 (0.81-1.22)	0.950	0.94 (0.78-1.13)	0.490
BMI (kg/m2)						
<25	0.90 (0.79-1.02)	0.089	1.02 (0.89-1.16)	0.790	0.98 (0.87-1.10)	0.710
≥25	0.98 (0.80-1.21)	0.860	0.89 (0.72-1.10)	0.290	0.99 (0.82-1.19)	0.910
Hypertension						
No	**0.84 (0.73-0.96)**	**0.013**	1.11 (0.96-1.29)	0.160	1.05 (0.92-1.20)	0.490
Yes	1.07 (0.90-1.28)	0.460	0.83 (0.69-1.00)	0.044	0.89 (0.75-1.05)	0.170
Diabetes mellitus						
No	0.93 (0.83-1.04)	0.230	0.99 (0.88-1.11)	0.840	0.99 (0.89-1.10)	0.890
Yes	0.68 (0.44-1.04)	0.071	0.85 (0.57-1.28)	0.440	0.95 (0.66-1.36)	0.770
Cardiovascular disease						
No	0.89 (0.80-1.00)	0.049	0.99 (0.88-1.12)	0.920	0.99 (0.89-1.11)	0.910
Yes	**1.59 (1.03-2.44)**	**0.033**	0.76 (0.49-1.18)	0.210	1.07 (0.74-1.55)	0.720
Gleason score						
≤7	0.90 (0.79-1.02)	0.094	1.00 (0.88-1.14)	0.960	0.96 (0.85-1.08)	0.460
≥8	0.96 (0.81-1.13)	0.590	0.93 (0.78-1.10)	0.400	1.02 (0.88-1.19)	0.790
Extracapsular extension						
No	0.89 (0.78-1.00)	0.058	0.98 (0.86-1.11)	0.700	1.01 (0.90-1.14)	0.860
Yes	0.99 (0.84-1.17)	0.890	0.95 (0.80-1.14)	0.600	0.89 (0.76-1.04)	0.130

Supplementary Table 4 Continued. Stratified analysis for associations between genetic polymorphisms in homocysteine removal genes and PCa risk by log-additive genetic model in Han Chinese men

Seminal vesicle invasion						
No	0.91 (0.81-1.02)	0.110	0.98 (0.87-1.11)	0.750	0.99 (0.88-1.10)	0.810
Yes	0.97 (0.80-1.17)	0.750	0.92 (0.75-1.13)	0.420	0.92 (0.77-1.11)	0.380
Positive surgical margin						
No	0.91 (0.81-1.02)	0.110	0.98 (0.87-1.11)	0.780	0.99 (0.89-1.10)	0.820
Yes	0.91 (0.73-1.13)	0.400	0.95 (0.75-1.20)	0.680	0.94 (0.76-1.15)	0.540
Lymph node involvement						
No	0.90 (0.81-1.01)	0.070	0.97 (0.87-1.09)	0.620	0.99 (0.89-1.10)	0.820
Yes	1.05 (0.78-1.41)	0.740	1.05 (0.77-1.44)	0.740	0.91 (0.68-1.21)	0.520

OR, odds ratio; 95%CI, 95% confidence interval.

[a]Adjusted for age, BMI, hypertension, diabetes mellitus, and cardiovascular disease in multivariant logistic regression models.

Supplementary Table 5. Genotypes of surveyed SNPs in cell lines

Cell lines	rs28372871	rs1131450	rs1805087	rs326119	rs1801394	rs2850144
LNCaP	TT	GG	AA	AA	AA	CC
PC3	TT	GG	AA	AA	AA	CC

Supplementary Table 6. DNA sequence of all used primer pairs

**Primer Name**	**Sequence (5'→3')**	**Purpose**
rs28372871-F	AACGCCTACTACAACCCTAAAA	PCR
rs28372871-R	AGTTCTGCGCTCAATCTATCC	PCR
rs1131450-F	CATGCCATTCTCCTGCCTCA	PCR
rs1131450-R	TGCCCACTTGTCCAACTCC	PCR
rs1805087-F	TTTCAGTGTTCCCAGCTGTTAGAT	PCR
rs1805087-R	AAACTAGGATCATAAAAAACAGTCACATT	PCR
rs326119-F	GCCTTTGGCTTTGGTGTCC	PCR
rs326119-R	TTCCGCAAGAGATAAGCAGTAGT	PCR
rs1801394-F	GGAAACACAGATTCAAGCCCAA	PCR
rs1801394-R	CCCAACCAAAATTCTTCAAAGC	PCR
rs2850144-F	CAGGCTGGCTGAAGGCTCT	PCR
rs2850144-R	CGCAGAACAGTCGCCTTG	PCR
rs28372871-typing	CGGCTGCGAGGAGCTCG	Genotyping
rs1131450-typing	AAATTAGCCGGGTGTGGTGG	Genotyping
rs1805087-typing	CACTTACCTTGAGAGACTCATAATGG	Genotyping
rs326119-typing	GTTCATTCACCGAAAGCCAAG	Genotyping
rs1801394-typing	AAAGGCCATCGCAGAAGAAAT	Genotyping
rs2850144-typing	CGGCCCGCGCAGCTCCGCCG	Genotyping
MTR-promter -F	ATGGCGACGCGTCGCCATCTCATTCCTCCCTCCCTTCTTT	Construct
MTR-promter -R	ACTGGAAGATCTTCCAGTCCTTGGTGTCGGCCTAGCAG	Construct
MTR-UTR -F	CACAACTCGAGCAGAACTCCCTTTGGCAAAAGGCAT	Construct
MTR-UTR -R	AAGGATCCCTTCATTTGTTCCTCCCTTGCTTCT	Construct
MTR-mRNA-F	CGCAACCCGAAGGTCTGAA	RT-qPCR
MTR-mRNA-R	TTCTTCGTTTAGCTTCTCCCG	RT-qPCR
GAPDH-mRNA-F	GAAGGTGAAGGTCGGAGTC	RT-qPCR
GAPDH-mRNA-R	GAAGATGGTGATGGGATTTC	RT-qPCR
